# A Resected Case of Hepatic Angiomyolipoma with Tumor Hemorrhage and Undetectable Fat Causing Diagnostic Difficulty

**DOI:** 10.70352/scrj.cr.25-0240

**Published:** 2025-07-08

**Authors:** Yumeo Tateyama, Yuichi Yamazaki, Ayaka Katayama, Tatsuma Murakami, Hiroki Tojima, Kenichiro Araki, Takaaki Sano, Ken Shirabe, Toshio Uraoka

**Affiliations:** 1 Department of Gastroenterology and Hepatology, Gunma University Graduate School of Medicine, Maebashi, Gunma, Japan; 2 Department of Diagnostic Pathology, Gunma University Graduate School of Medicine, Maebashi, Gunma, Japan; 3 Division of Hepatobiliary and Pancreatic Surgery, Department of General Surgical Science, Gunma University Graduate School of Medicine, Maebashi, Gunma, Japan

**Keywords:** hepatic angiomyolipoma, imaging, laparoscopic hepatectomy, mesenchymal tumor, preoperative diagnosis, tumor hemorrhage

## Abstract

**INTRODUCTION:**

Hepatic angiomyolipoma (AML) is a rare, benign mesenchymal tumor with variable imaging features, often complicating differentiation from malignancy. We report a case of hepatic AML that showed progressive enlargement due to intratumoral hemorrhage, without detectable fat on imaging.

**CASE PRESENTATION:**

A woman in her 70s with no history of chronic liver disease had previously undergone surgery for lung adenocarcinoma and for localized nodular hyperplasia of the liver. Routine follow-up imaging revealed an enlarging liver mass in the right hepatic lobe, leading to her referral for further evaluation. Tumor markers were within normal ranges, and liver function remained intact. Non-contrast CT showed a low-attenuation nodule, and contrast-enhanced CT demonstrated ring-like peripheral enhancement with a hypovascular center. Magnetic resonance imaging showed low signal on T1-weighted images and high signal on T2-weighted images, with no signal loss in opposed-phase imaging. Ultrasonography demonstrated a low echogenicity within the tumor and high echogenicity in the surrounding area, with no clear contrast effect. The preoperative diagnosis suggested either a hematoma or a necrotic nodule. Given the progressive growth and inconclusive imaging, malignancy could not be excluded. A laparoscopic hepatectomy was performed for definitive diagnosis. The resected tumor was a 2.3 × 2.0 × 1.4 cm well-demarcated, light brown mass with areas of hemorrhage and cystic change. Histopathology confirmed hepatic AML with tumor hemorrhage and extramedullary hematopoiesis.

**CONCLUSIONS:**

Hepatic AML may exhibit progressive growth despite lacking typical imaging features such as intratumoral fat or vascularity, making preoperative diagnosis difficult. In cases where malignancy cannot be ruled out, surgical resection should be considered after careful evaluation of both benign and malignant possibilities.

## Abbreviations

AFP
: alpha-fetoprotein
AML
: angiomyolipoma
CA19-9
: carbohydrate antigen 19-9
CEA
: carcinoembryonic antigen
HCC
: hepatocellular carcinoma
HMB-45
: human melanoma black 45
HU
: Hounsfield unit
ICG
: indocyanine green
PEComa
: perivascular epithelioid cell tumor
PIVKA-II
: protein induced vitamin K absence or antagonist II
S7
: segment 7
αSMA
: alpha-smooth muscle actin

## INTRODUCTION

AML is a rare, benign mesenchymal tumor classified as a PEComa. Its imaging features vary depending on the proportions of blood vessels, smooth muscle, and fat, making differentiation from malignancies, such as HCC, particularly difficult when fat content is minimal.^1–4)^

Although typically benign, hepatic AMLs larger than 5 cm may rupture and cause hemorrhage, underscoring the importance of early and accurate diagnosis^2)^. Moreover, their imaging findings can overlap with those of aggressive liver tumors, necessitating a combined diagnostic approach involving imaging, histopathology, and immunohistochemical analysis.^5,6)^

Here we report a case of hepatic AML without visible fat or hypervascularity on imaging. Progressive tumor growth and hematoma raised suspicion of malignancy, leading to laparoscopic hepatectomy for definitive diagnosis and treatment.

## CASE PRESENTATION

**Fig. 1:**
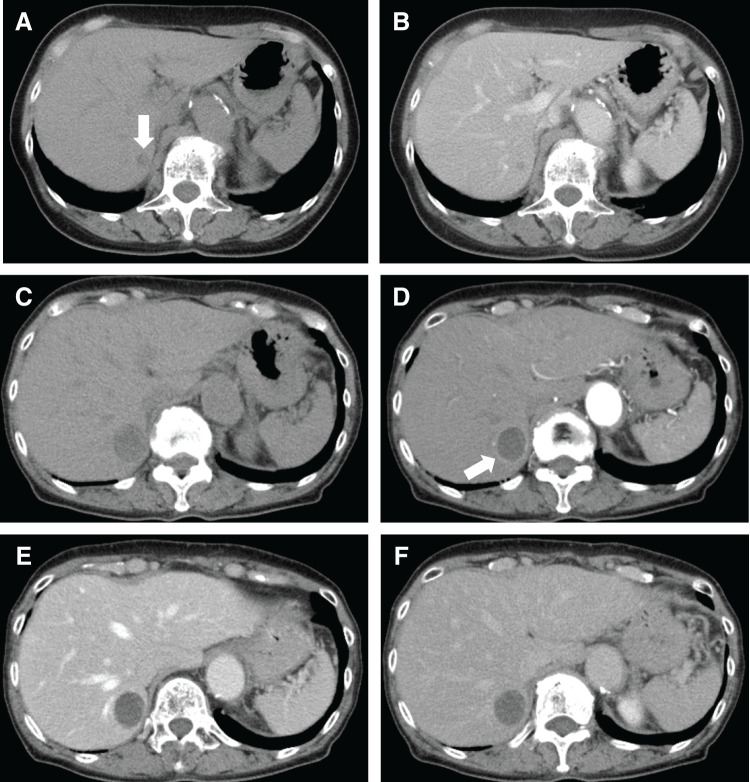
Abdominal CT findings. CT findings 1 year prior to the visit to our facility revealed a small low-attenuation nodule measuring 6 mm in diameter (arrow) (**A**, non-contrast and **B**, contrast-enhanced). (**C**) A non-contrast CT scan before surgery revealed a low-attenuation nodule measuring 20 mm in diameter. A contrast-enhanced CT scan showed a ring-like contrast enhancement with an oligemic interior (arrow) (**D**, arterial phase, **E**, portal phase, and **F**, late phase).

**Fig. 2:**
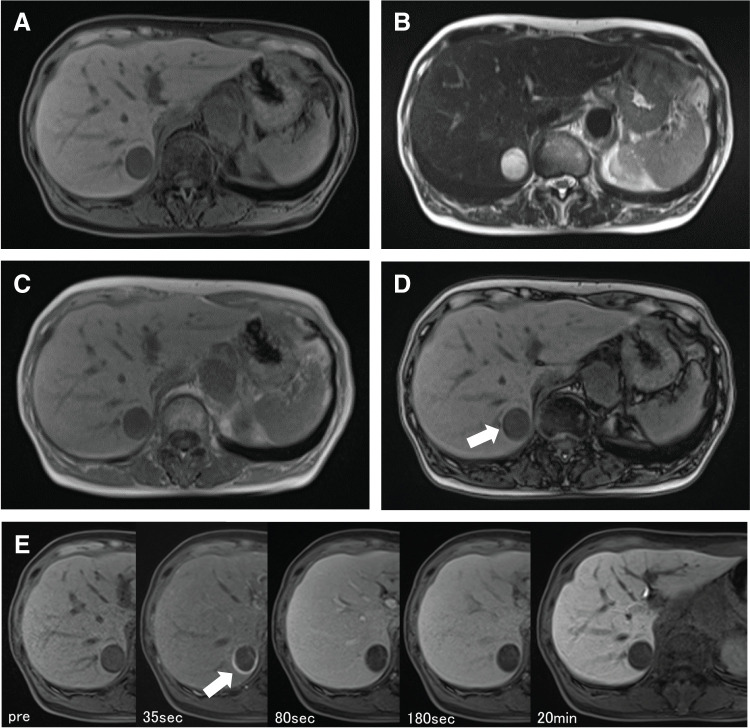
Abdominal MRI findings on admission before surgery. MRI findings showed (**A**) a low T1 signal and (**B**) a high T2 signal tumor, and (**C**, **D**) T1 in-phase and out-of-phase findings did not indicate fat throughout the tumor, although a slight signal drop was observed at the tumor margin (arrow). (**E**) A contrast-enhanced MRI also demonstrated a ring-like contrast enhancement in the arterial phase (arrow), but with an oligemic interior.

A woman in her 70s, without chronic liver disease, had undergone surgery for lung adenocarcinoma 2 years earlier and for localized nodular hyperplasia of the liver 12 years ago. Routine imaging revealed an enlarging mass in the right hepatic lobe, prompting referral to our facility. Throughout the course of her follow-up, she remained asymptomatic, with no abdominal pain, and liver function tests were normal. Tumor markers, such as AFP, PIVKA-II, CEA, and CA19-9, were within normal limits (**Table 1**). A CT scan 1 year before referral showed a 6 mm hypodense nodule in S7 of the liver without contrast enhancement. However, since this was not a dynamic CT, arterial-phase enhancement was not assessed. While the nodule appeared hypodense, it could not be definitively characterized as fat-rich. No additional imaging studies, such as abdominal ultrasound or MRI, were performed at that time (**Fig. 1A** and **1B**). At the time of referral, non-contrast CT revealed a low-attenuation nodule measuring 20 mm in diameter in the same location, and contrast-enhanced CT showed ring-like enhancement with an oligemic interior (**Fig. 1C** and **1F**). Three months later, the CT indicated that the mass at the same site had increased in size to 2.7 cm in diameter. MRI showed low signal intensity on T1-weighted images and high signal intensity on T2-weighted images (**Fig. 2A** and **2B**). On opposed-phase imaging, a slight signal drop was observed at the tumor margin, but no significant signal loss was seen throughout the tumor, suggesting the absence of clear fat content (**Fig. 2C** and **2D**). Contrast-enhanced MRI revealed ring-like enhancement during the arterial phase, with an oligemic center and hypervascularity limited to the periphery (**Fig. 2E**).

**Table 1:** Laboratory findings before surgery

Hematology			Biochemistry			Tumor Marker		
Hb	14.6	g/dL	TP	6.8	g/dL	AFP	1.8	ng/mL
RBC	453 × 10^4^	/μL	Alb	4.1	g/dL	PIVKA-II	13.9	mAU/L
Ht	42.3	%	T-Bil	0.8	mg/dL	CEA	1.8	ng/mL
WBC	4300	/μL	AST	21	IU/L	CA19-9	10	U/mL
PLT	20.5 × 10^4^	/μL	ALT	16	IU/L	SCC	0.5	ng/mL
			LDH	212	IU/L	CYFRA	1.7	ng/mL
**Coagulation Test**			ALP	91	IU/L	CA125	10	ng/mL
PT%	93	%	γGTP	13	IU/L			
PT-INR	1.02		ChE	299	IU/L	ICG R15	13.3	%
APTT	29.5	sec	Amy	74	IU/L			
Fbg	256	mg/dL	BUN	20	mg/dL			
FDP	2.5	ug/mL	Cre	0.56	mg/dL	**Virus Marker**		
D-dimer	0.5	ug/mL	Na	141	mEq/L	HBsAg	(−)	
			Cl	105	mEq/L	HBsAb	(−)	
			K	3.9	mEq/L	HBcAb	(−)	
			Ca	9.4	mg/dL	HCVAb	(−)	
			CRP	0.03	mg/dL			
			BS	95	mg/dL			

AFP, alpha-fetoprotein; CA125, carbohydrate antigen 125; CA19-9, carbohydrate antigen 19-9; CEA, carcinoembryonic antigen; CYFRA, Cytokeratin 19 fragment; HBcAb, hepatitis B core antibody; HBsAb, hepatitis B surface antibody; HBsAg, hepatitis B surface antigen; HCVAb, hepatitis C virus antibody; ICG R15, indocyanine green 15-minute retention rate; PIVKA-II, protein induced vitamin K absence or antagonist II; SCC, squamous cell carcinoma associated antigen

**Fig. 3:**
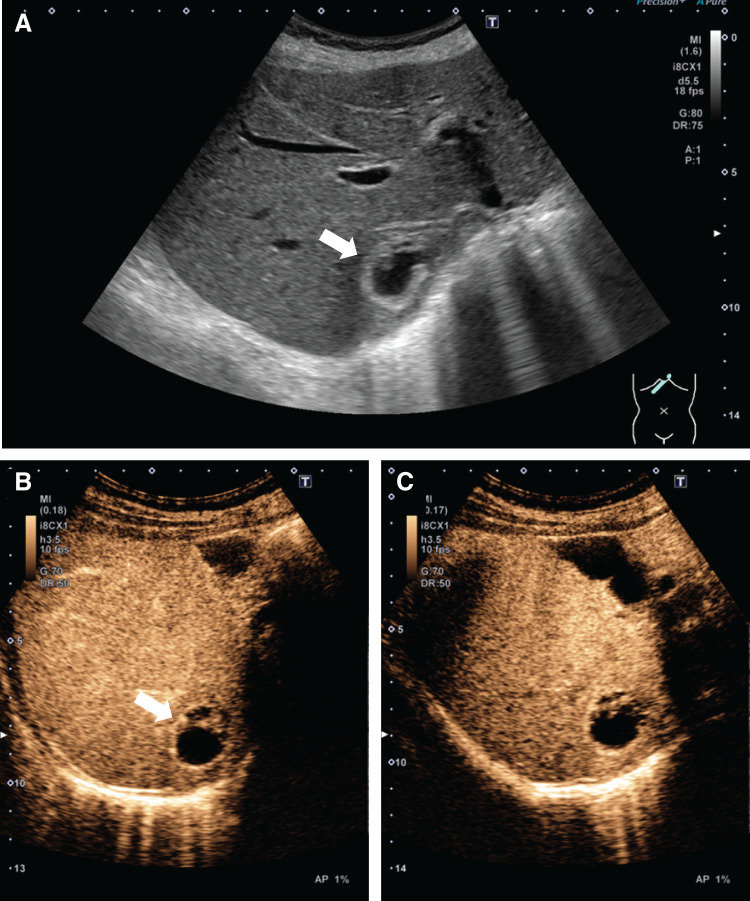
Abdominal ultrasonography findings before surgery. (**A**) B-mode ultrasonography showed homogeneous hypoechoic pattern with hyperechoic rim and septum-like structures (arrow). Contrast-enhanced ultrasonography using Sonazoid (GE Healthcare, Tokyo, Japan) demonstrated (**B**) enhancement only at the tumor periphery and septum-like structures (arrow), and (**C**) no clear defect in the Kupffer phase.

Positron emission tomography showed no significant tracer uptake within the tumor, suggesting a low likelihood of malignancy. On ultrasonography, the tumor appeared as a hypoechoic mass, while the tumor margins and septum-like structures exhibited hyperechogenicity (**Fig. 3A**). Contrast-enhanced ultrasonography revealed enhancement only at the tumor periphery and septum-like structures, with no clear defect observed in the Kupffer phase (**Fig. 3B** and **3C**). The septum-like structures within the tumor were not clearly delineated on CT or MRI.

**Fig. 4:**
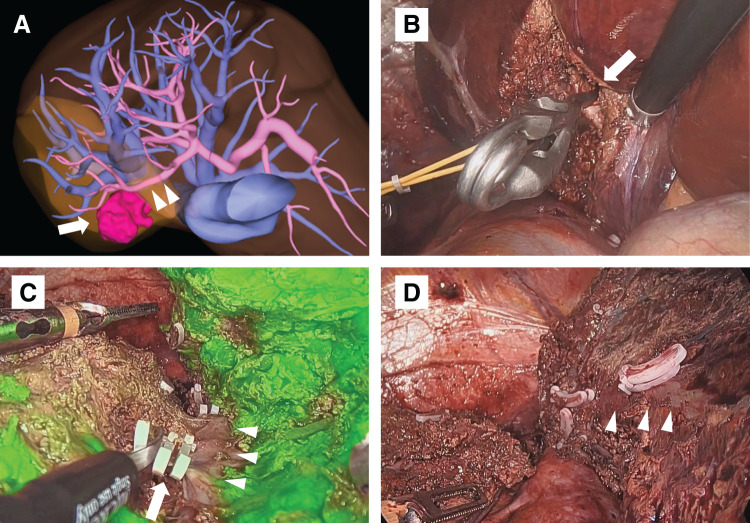
Operative findings of laparoscopic S7 segmentectomy. (**A**) Preoperative 3D reconstruction image. The tumor (arrow) is located in segment 7 (S7), and the Glissonean branch of S7 is indicated (arrowheads). (**B**) The Glissonean branch of S7 (arrow) is isolated using an intrahepatic approach and clamped by bulldog forceps. (**C**) Parenchymal transection is performed along the intersegmental plane, which is identified using the ICG-negative staining method. The right hepatic vein (arrowheads) is exposed, and the V7 branch (arrow) is divided. (**D**) Post-transection surface. The right hepatic vein (arrowheads) is clearly exposed. ICG, indocyanine green

The preoperative differential diagnosis included a hematoma, a necrotic nodule, or a malignancy such as HCC or cystadenocarcinoma, given the observed tumor growth. Although a tumor biopsy was considered, the high proportion of fluid components and the small amount of solid tumor tissue increased the risk of sampling error. Additionally, in cases of HCC or cystadenocarcinoma, there was a high risk of rebleeding or tumor seeding. Therefore, laparoscopic S7 segmentectomy was performed for both definitive diagnosis and treatment. The surgical procedure is shown in **Fig. 4**. Briefly, the Glissonean branch of S7 was isolated using an intrahepatic approach, and liver parenchymal transection was then performed along the intersegmental plane using the ICG negative staining method.^7)^ The operation time was 235 minutes, and the estimated blood loss was 23 mL. The patient was discharged on postoperative day 14 without complications.

**Fig. 5:**
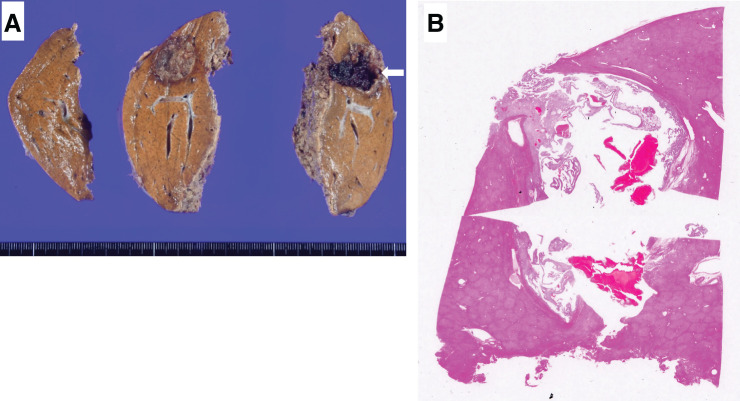
Macroscopic findings and microscopic findings (loupe image). In macroscopic findings (**A**) after formalin fixation, the tumor was a well-demarcated, solid mass measuring approximately 2.3 × 2.0 × 1.4 cm, light brown in color with areas of hemorrhage and cystic change (arrow). (**B**) Hematoxylin and eosin staining (loupe image) showed that the solid portions of the tumor were distributed at the margins, while the central part was cavitated and filled with clotted blood.

Macroscopically, the tumor was a well-demarcated, solid mass measuring approximately 2.3 × 2.0 × 1.4 cm, with a light brown color and areas of hemorrhage and cystic change (**Fig. 5A**).

**Fig. 6:**
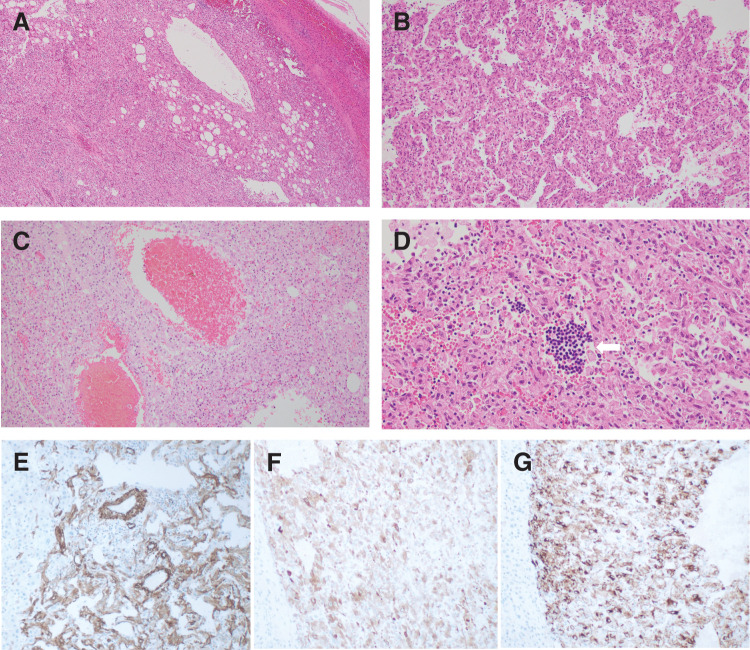
Microscopic and immunostaining findings. (**A**) Hematoxylin and eosin staining showed that the tumor was composed of adipose tissue, blood vessels, and smooth muscle tissue (× 100). Among these smooth muscle-like cells, (**B**) sinusoidal-like vascular spaces on the lumen side and (**C**) thick-walled muscular vessels were observed (× 200). (**D**) Foci of extramedullary hematopoiesis (arrow) were present within the tumor (× 200). Immunostaining was positive for (**E**) αSMA (× 100), (**F**) Melan A (× 100), and (**G**) HMB-45 (× 100). HMB-45, human melanoma black 45; αSMA, alpha-smooth muscle actin

Microscopically, the tumor was largely occupied by hematoma, with the solid components localized at the periphery, consisting of adipose tissue, blood vessels, and smooth muscle tissue, consistent with the characteristics of hepatic angiomyolipoma (**Fig. 5B** and **6A**). The solid components of the tumor were approximately composed of 10% adipose tissue, 90% smooth muscle tissue, and blood vessels. The tumor consisted of spindle-shaped to epithelioid cells with eosinophilic cytoplasm, typical of smooth muscle-like cells, proliferating in fascicular or sheet-like patterns. Among these smooth muscle-like cells, thick-walled muscular vessels, clusters of mature adipocytes without fibrous capsule (**Fig. 6A**), and sinusoid-like vascular spaces on the luminal side (**Fig. 6B**) were observed. In various regions of the tumor, hemorrhagic areas were evident, characterized by red blood cell accumulation, tissue dissociation, cystic degeneration, and lymphocyte infiltration (**Fig. 6C**). Additionally, foci of extramedullary hematopoiesis were present within the tumor (**Fig. 6D**). Immunohistochemical staining was positive for αSMA (**Fig. 6E**), Melan A (**Fig. 6F**), and HMB-45 (**Fig. 6G**), confirming the diagnosis of hepatic AML.^6,8)^

## DISCUSSION

A hepatic tumor lacking both fat and vascularity raised suspicion of malignancy. Due to its atypical imaging features and inconclusive diagnosis, laparoscopic hepatectomy was performed. Histological examination confirmed hepatic AML with hemorrhage. This case highlights the importance of considering AML in the differential diagnosis of atypical hepatic tumors with progressive growth.

Hepatic AML is a rare mesenchymal tumor composed of varying proportions of blood vessels, smooth muscle, and fat, with any component potentially being dominant. Typically, hepatic AML exhibits characteristic imaging findings, such as homogeneous hyperechogenicity on ultrasound, low attenuation on non-contrast CT (with values below −20 HU if fat is present), and high signal intensity on both T1- and T2-weighted MRI sequences.^2,3)^ Furthermore, hepatic AMLs commonly demonstrate hypervascularity and tumor enhancement across various imaging modalities. Recognizing these features is crucial for preoperative diagnosis, particularly the presence of fat, which is a key indicator distinguishing hepatic AML from other hepatic tumors^4,5)^.

However, the fat content in hepatic AMLs varies significantly between cases, ranging from 5% to over 50%, complicating imaging interpretation.^8)^ Fat-poor hepatic AMLs pose a significant diagnostic challenge as they lack the typical imaging characteristics associated with fat-rich hepatic AMLs. The absence of substantial fat content may cause the tumor to resemble malignant hepatic lesions, such as hepatocellular carcinoma or metastatic tumors.^8,9)^ In this case, preoperative CT and MRI revealed no detectable fatty component, and contrast studies showed oligemic features in most of the tumor, making definitive diagnosis based on imaging alone difficult.

This case exhibited atypical imaging features that can be attributed to its underlying histopathological composition, particularly the peripheral localization of tumor components and extensive intratumoral hemorrhage. Histopathological examination of the resected tumor provided further insights into these diagnostic challenges. Hemorrhage was present within the tumor, with the hepatic AML components—blood vessels, smooth muscle, and fat—localized exclusively at the periphery. The peripheral enhancement observed on contrast-enhanced CT, MRI, and ultrasonography was attributed to this peripheral tumor localization. The mild signal drop at the tumor periphery on opposed-phase MRI and the hyperechoic rim observed on ultrasound may have suggested fat content. This interpretation was supported by histological findings, which confirmed that the solid components were confined to the periphery of the tumor and included approximately 10% adipose tissue. This adipose tissue corresponded to the peripheral areas of signal alteration on imaging, thereby explaining the subtle fat-related imaging features.

At a fat content of approximately 10%, opposed-phase MRI has been reported to demonstrate a sensitivity of around 90% and a specificity of approximately 95%, while abdominal ultrasonography shows a similar sensitivity of about 90%, but a lower specificity of around 70%.^10)^ These imaging findings are therefore considered to indicate the presence of mild fat content. The histological characteristics, where much of the tumor was composed of hematoma with the solid AML components restricted to the edges, likely contributed to the atypical imaging findings and the difficulty in establishing a preoperative diagnosis.

Preoperative imaging suggested that the tumor was predominantly composed of an enlarging hematoma or necrotic tissue, but malignancy such as HCC or cystadenocarcinoma could not be excluded. When malignancy, including cystadenocarcinoma, is suspected, tumor biopsy is often avoided due to the high risk of bleeding or tumor seeding.^11,12)^ In this case, malignancy could not be ruled out based on imaging alone. Given the risks of bleeding and tumor seeding associated with biopsy, surgical resection was performed both for diagnostic confirmation and treatment.

Hemorrhage within hepatic AML is not uncommon, and in some cases, tumor hemorrhage can lead to rapid growth, necessitating surgical intervention.^13,14)^ Since hepatic AML is a rare entity, its risk factors remain poorly defined. However, large tumors exceeding 5 cm, tumors with a high proportion of vascular components, and pregnancy—reported as a risk factor for renal AML—may contribute to tumor hemorrhage.^2,15)^ In this case, although the tumor measured only 2 cm at the time of diagnosis and showed no rapid growth of the AML component, pathological examination of the resected tumor revealed that approximately 90% consisted of vascular and smooth muscle components, with half of the vascular components being sinusoid-like vascular spaces, which may have contributed to the hemorrhage.

Extramedullary hematopoiesis in hepatic AML is considered a characteristic finding. Unlike renal AML, hepatic AML may occasionally be accompanied by extramedullary hematopoiesis. Western reports indicate that extramedullary hematopoiesis has been observed in almost 50% of hepatic AML cases.^16,17)^ The presence of extramedullary hematopoiesis may serve as an important diagnostic feature of hepatic AML. Goodman and Ishak^16)^ even proposed that cases containing this component should be classified as “angiomyomyelolipoma.” In the present case, extramedullary hematopoiesis was identified histopathologically and was a useful finding in confirming the diagnosis of hepatic AML.

## CONCLUSIONS

Hepatic AML may demonstrate progressive growth despite lacking typical imaging features such as intratumoral fat or vascularity, making preoperative diagnosis challenging. In such cases, where malignancy cannot be confidently excluded, surgical resection should be considered after thorough evaluation of both benign and malignant possibilities.

## DECLARATIONS

### Funding

No significant financial support or funding was received for this study that could have influenced its outcome.

### Authors’ contributions

YT drafted the manuscript and provided the original figures.

YY critically reviewed and revised the manuscript.

KA reviewed the manuscript and provided the original figure about surgical procedure.

TM, HT, KS, and TU reviewed the manuscript.

AK and TS performed the pathological diagnosis.

All authors read and approved the final manuscript.

### Availability of data and materials

Data sharing is not applicable to this article.

### Ethics approval and consent to participate

Ethics approval is not applicable because this is a case report.

### Consent for publication

Written informed consent was obtained from the patient for the publication of this case report.

### Competing interests

The authors declare that they have no competing interests.

### Generative AI and AI-assisted technologies in the writing process

During the preparation of this work the author used ChatGPT-4o in order to improve language and readability. After using this service, the author reviewed and edited the content as needed and takes full responsibility for the content of the publication.
